# Multiple actions of lysophosphatidic acid on fibroblasts revealed by transcriptional profiling

**DOI:** 10.1186/1471-2164-9-387

**Published:** 2008-08-14

**Authors:** Catelijne Stortelers, Ron Kerkhoven, Wouter H Moolenaar

**Affiliations:** 1 Division of Cellular Biochemistry and Centre for Biomedical Genetics, The Netherlands Cancer Institute, Plesmanlaan 121, 1066 CX Amsterdam, The Netherlands; 2 Central Microarray Facility, The Netherlands Cancer Institute, Plesmanlaan 121, 1066 CX Amsterdam, The Netherlands; 3 Ablynx, Technologiepark 4, 9052 Ghent/Zwijnaarde, Belgium

## Abstract

**Background:**

Lysophosphatidic acid (LPA) is a lipid mediator that acts through specific G protein-coupled receptors to stimulate the proliferation, migration and survival of many cell types. LPA signaling has been implicated in development, wound healing and cancer. While LPA signaling pathways have been studied extensively, it remains unknown how LPA affects global gene expression in its target cells.

**Results:**

We have examined the temporal program of global gene expression in quiescent mouse embryonic fibroblasts stimulated with LPA using 32 k oligonucleotide microarrays. In addition to genes involved in growth stimulation and cytoskeletal reorganization, LPA induced many genes that encode secreted factors, including chemokines, growth factors, cytokines, pro-angiogenic and pro-fibrotic factors, components of the plasminogen activator system and metalloproteases. Strikingly, epidermal growth factor induced a broadly overlapping expression pattern, but some 7% of the genes (105 out of 1508 transcripts) showed differential regulation by LPA. The subset of LPA-specific genes was enriched for those associated with cytoskeletal remodeling, in keeping with LPA's ability to regulate cell shape and motility.

**Conclusion:**

This study highlights the importance of LPA in programming fibroblasts not only to proliferate and migrate but also to produce many paracrine mediators of tissue remodeling, angiogenesis, inflammation and tumor progression. Furthermore, our results show that G protein-coupled receptors and receptor tyrosine kinases can signal independently to regulate broadly overlapping sets of genes in the same cell type.

## Background

Lysophosphatidic acid (LPA; monoacyl-glycerol-3-phoshate) is a lipid mediator that stimulates the proliferation, migration and survival of many cell types [1]. LPA acts through at least five distinct G protein-coupled receptors (GPCRs), termed LPA_1–5_, which show overlapping signaling properties and tissue distribution [2,3]. LPA signaling has been implicated in a great variety of biological processes, ranging from embryonic development to wound healing and tumor progression [1-5]. This multitude of activities is consistent with the broad distribution of LPA receptors and their coupling to multiple G proteins. LPA is produced from more complex lysophospholipids by a secreted lysophospholipase D known as autotaxin (ATX), originally identified as an 'autocrine motility factor' for tumor cells (for review see ref. [3]). ATX is essential for vascular development [6,7] and in addition promotes tumor aggressiveness and angiogenesis [8]. This strongly suggests that LPA is a key pro-angiogenic factor during development and a signifcant effector of cancer progression in the stroma-tumor microenviroment.

Although LPA-induced signaling pathways and cellular responses have been extensively analyzed over the years [1], it remains unknown how LPA affects global gene expression in its target cells. Gene expression analysis may uncover previously unknown activities of LPA, lead to a better understanding of GPCR signaling in general, and help to predict the behavior of cells in an LPA-enriched environment.

In this report, we analyze the global transcriptional response to LPA in mouse embryo fibroblasts (MEFs). Fibroblasts are abundant mesenchymal cells in the stroma of many tissues and organs where they regulate epithelial-mesenchymal interactions during development, tissue regeneration and tumor progression [9,10]. LPA has long been known to stimulate the proliferation and migration of fibroblasts [1,11], while excessive LPA signaling in these cells can lead to fibrosis [12]. The present study identifies many novel LPA-regulated genes and shows especially that LPA commits fibroblasts, at the transcriptional level, to create a microenvironment that supports tissue remodeling, leukocyte recruitment, angiogenesis and tumor progression. Since fibroblasts are also responsive to epidermal growth factor (EGF), acting on its cognate receptor tyrosine kinase, we examined in addition to what extent the LPA- and EGF-induced expression patterns overlap.

## Results and Discussion

### Characterization of MEFs

**Figure 1:**
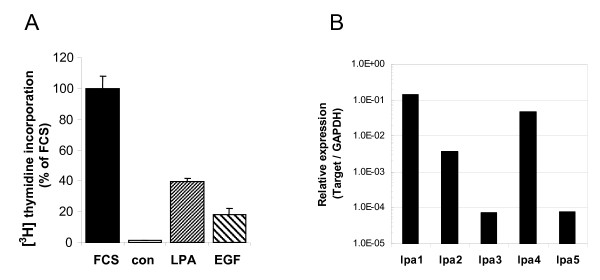
**Characterization of MEFs**. **(A) **Mitogenic responsiveness of MEFs as measured by [^3^H]thymidine incorporation. Serum-deprived cells ("control") were treated with fetal calf serum (FCS; 10%), LPA (10 μM) or EGF (25 ng/ml). The response to FCS was set at 100%. Bars represent means ± SD (N = 3). **(B) **LPA receptor expression in MEFs. Expression levels were determined by qPCR using the GAPDH gene for normalization. Expression levels of LPA_3 _and LPA_5 _are negligible.

We examined the mitogenic response and LPA receptor expression profile of immortalized mouse embryonic fibroblasts (MEFs). Stimulation of serum-deprived, nearly confluent MEFs with saturating doses of LPA (5 μM) resulted in a significant increase in DNA synthesis to about 40% the level induced by 10% serum. EGF (50 ng/ml) was somewhat less efficacious than LPA in stimulating DNA synthesis (Figure 1A). LPA signals via at least five distinct GPCRs (termed LPA_1–5_) that couple to multiple G proteins, including G_i/o_, G_q/11 _and G_12/13_. Quantitative PCR analysis showed that our MEFs co-expressed LPA_1_, LPA_2 _and LPA_4_, with LPA_1 _and LPA_4 _being the predominant transcripts (Figure 1B). Since MEFs do not express ATX (encoded by *Enpp2*; data not shown), autocrine LPA signaling is not operative in our cell system.

### The global transcriptional response to LPA

**Figure 2:**
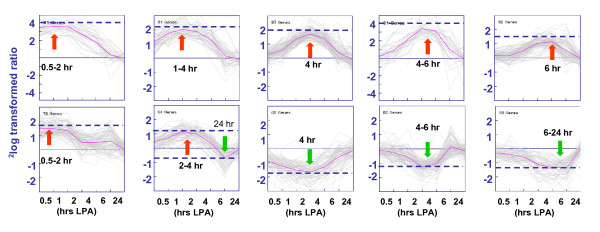
**Global transcriptional response of MEFs to LPA**. Gene expression patterns induced by LPA (5 μM) clustered into 10 different classes (K-means clustering analysis; N = 10). Clusters were generated based on the time point of maximal induction. Data indicate ^2^log ratios of transcripts with p ≤ 0.001 in at least two consecutive time points (resulting in 633 selected transcripts). Each column represents one time point of LPA treatment; the last column shows the expression in non-synchronized MEFs. The time point or period of maximal induction (red) or reduction (green) is indicated in each cluster by an arrow and the respective hour. Solid lines (pink) indicate the median temporal pattern of expression; dotted lines indicate the median level for each cluster. See additional files 2 and 3 for details of the individual clusters.

**Figure 3:**
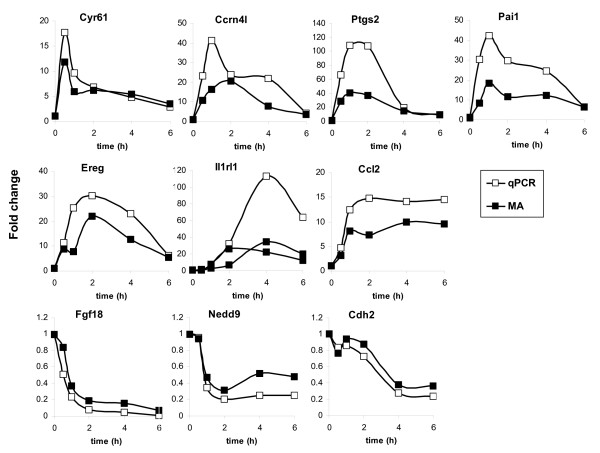
**Validation of microarray data**. Comparison of the relative expression levels of genes selected from different clusters, as determined by microarray (closed symbols) and qPCR (open symbols). qPCR data were normalized to HPRT mRNA concentration and plotted relative to the level at time zero. Data are presented as means ± SD of duplicate experiments. Note that the qPCR assays generally yielded higher mRNA values than the microarray analysis. See also the correlation plot in additional file 6.

We examined the temporal program of gene expression in serum-starved MEFs treated with LPA (5 μM, i.e. about the normal concentration in serum [13]). Total RNA was isolated at different time points after LPA stimulation (0–24 hrs). Global transcription profiles were determined using oligonucleotide microarrays containing 31,770 mouse transcripts. Amplified RNA of the treated samples was matched with the untreated control and hybridized in duplicate with reversal of the Cy3 and Cy5 dyes; the normalized Cy5/Cy3 ratios were combined and used for further analysis. We selected genes that were significantly regulated (p < 0.01) at two or more consecutive time points or in replicate measurements, which yielded 1508 LPA-regulated genes (see additional file 1: complete dataset). The entire dataset has been deposited in the EBI/ArrayExpress database (see Methods). We restricted our data set to genes that were induced by >1.7-fold at two or more time points and grouped them according to the temporal profile of gene induction using K-means clustering. This resulted in ten clusters, each containing genes that show similarly shaped waves of transcription (Figure 2). Seven clusters contained genes that were upregulated by LPA (424 transcripts), whereas three other clusters mainly comprised the down-regulated genes (209 transcripts) (Figure 2; for details see additional files 2 and 3). The genes that were most strongly regulated at different time points are listed in additional file 4 (Table 1: upregulated genes; Table 2: downregulated genes). The microarray results were validated by examining the expression of 22 representative genes from different clusters using real-time PCR (Figure 3). The -fold inductions of expression generally were higher in the qPCR assays than in the corresponding microarray experiments, reflecting the different sensitivities of both methods (see additional file 5: correlation plot of all data points). Gene ontology analysis revealed that LPA regulated the expression of genes in multiple functional categories that mostly corresponded to the different gene clusters (see additional file 6).

### Regulation of genes associated with growth regulation and cytoskeletal reorganization

#### Upregulated genes

The immediate transcriptional response to LPA was characterized by >100 upregulated immediate-early genes (mRNA levels peaking at 0.5–1 hr) (Figure 2; see additional files 2 and 2/Table 1). This gene set contained various transcription factors associated with growth stimulation and cell cycle progression, notably genes encoding the AP-1 complex (*Fos, Fra1/FosL1, Jun, Atf3) *and other growth regulatory genes *(Egr1, Egr2*, *Klf6*, *Myc)*. Strong upregulation was also observed for *Ptgs2*, a highly inducible gene that encodes cyclooxygenase-2 (Cox-2) and has important roles in normal tissue homeostasis and inflammation. Upregulation of some of these immediate-early genes, including *Egr1*, *Jun, Myc *and *Ptgs2*, has earlier been found in LPA-treated MEFs and ovarian carcinoma cells [14,15]. LPA also induced the expression of genes encoding growth-regulatory protein kinases, including Sgk1, Dyrk3, Nuak2 and Map2k3 (Mek3). In addition to these 'forward-driving' genes, the early gene clusters contained various 'feedback regulators' known to turn off gene expression and/or attenuate prolonged signaling (see additional file 4: Table 1). Co-expression of both negative and positive regulators may be critical for the precise control of cell cycle progression. Among the feedback regulators induced by LPA were transcriptional repressors (*Ztbtb16*, *Lfrd1/Tis7*, *Pawr*), genes that promote the degradation of inducible mRNAs (*Zfp36, Nocturnin/Ccrnl2*) and a number of dual-specificity phosphatase (DUSP) genes, notably *Dusp1, Dusp5, Dusp6*, and *Dusp10*, whose products attenuate the activity of MAP kinases [16]. It is further of note that LPA induced a very robust upregulation of *Mig-6 *also known as *Errfi1 *(ErbB receptor feedback inhibitor 1; ~60-fold induction after 2 h). MIG-6 is a scaffold protein that interacts with the EGF receptor to inhibit its catalytic activity and all downstream signaling events [17,18]. By upregulating *Mig-6*, LPA may keep fibroblasts poised to prevent uncontrolled EGF receptor activation.

In addition to the above growth-regulatory genes, prominent upregulation was observed for genes whose products regulate the cytoskeleton, including various actin isoforms, vinculin and integrin alpha-subunits (*Itga5 *and *Itga6*), consistent with LPA's function as a regulator of cell shape and motility (see additional file 4: Table 1).

#### Downregulated genes

Strongest downregulation of gene expression was mostly observed at ~4–6 hrs after LPA stimulation, i.e. coincident with the mid-G1 phase of the cell cycle (Figure 2; see additional file 3). The most strongly down-regulated genes are listed in additional file 4 (Table 2); it comprises the transcription factor *Sox4*, recently identified as a mediator of metastasis [19], the transcriptional repressor *Slug *(*Snai2*), an inducer of the mesenchymal phenotype and a marker of malignancy [20], as well as genes that promote growth arrest. The latter set included the cell cycle inhibitors *Ccng2 *(encoding cyclin G2) and *Cdkn1b *(*p27Kip*), and the 'growth arrest-specific' genes *Gas1-3 *and *Ccn5*. The latter gene encodes a growth inhibitory matrix protein (CCN5) whose reduced expression promotes mesenchymal cell motility [21]. Other strongly downregulated genes were *Nedd9/Hef1 *and *Cdh2*. The Nedd9 scaffold is a downstream effector of focal adhesion kinase that may transduce integrin "inside-out" signaling to regulate cell-matrix adhesion and invasion [22,23], while *Cdh2 *encodes N-cadherin, a key regulator of intercellular adhesion strength in fibroblasts [24]. Through the coincident loss of N-cadherin, Nedd9 and CCN5, together with the upregulation of secreted metalloproteases (see below), LPA-stimulated fibroblasts may reduce their adhesive contacts and acquire a more motile and invasive phenotype.

### Induction of genes that encode secreted factors

**Figure 4:**
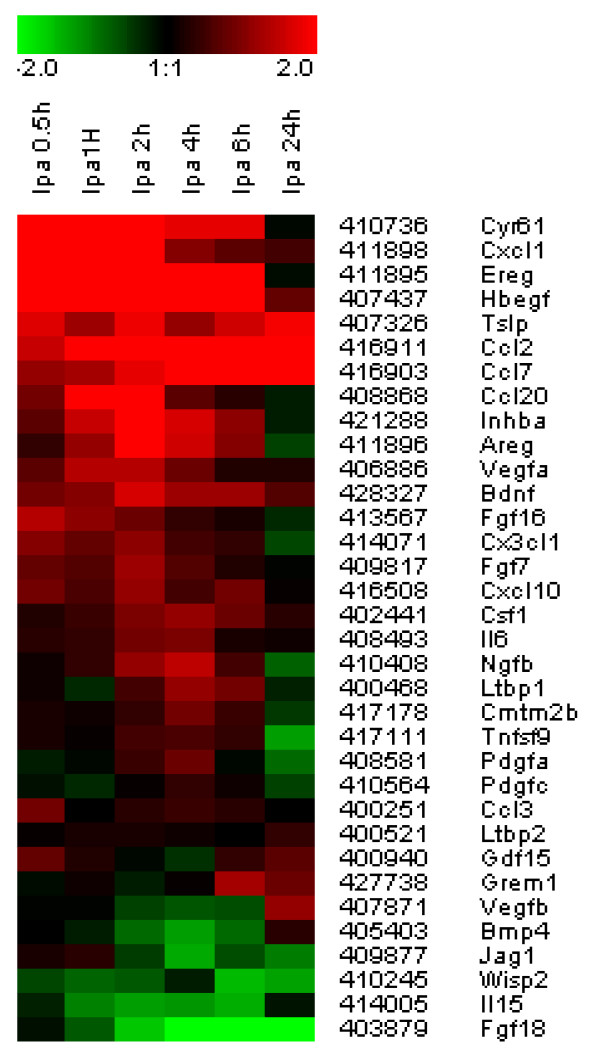
**Temporal expression pattern of LPA-regulated genes that encode secreted factors**. The heat map shows expression profiles of 34 selected genes as indicated. See also additional file 4 (Table 1).

Aside from genes associated with cell proliferation and motility, the immediate-early and early clusters contained many genes that encode paracrine factors such as chemokines, cytokines, mitogens and pro-angiogenic factors that are involved in inflammation, tissue remodeling and wound healing. Previous studies have shown that LPA induces the expression of the chemokine CXCL1/Gro-alpha and the cytokines IL-6 and IL-8 in ovarian cancer cells [25,26]. Figure 4 shows a heat map for 34 selected genes that encode secreted factors, with the most strongly induced genes at the top of the map. These include genes encoding CXCL1/Gro-alpha, IL-6, the EGF family members HB-EGF, epiregulin and amphiregulin, PDGF-A, CSF-1, VEGF-A and the 'pro-fibrotic' and vasoactive ligand endothelin-1 (*End1*). LPA also triggered robust upregulation of *Ccn1/Cyr61 *(peaking at 0.5 hr) and *Ccn2/Ctgf *(connective-tissue growth factor; peaking at 2 hrs). The *Ccn *genes encode matrix proteins that control cell attachment and migration, but also are important players in the pathogenesis of fibrosis [27].

Components of the urokinase-type plasminogen activator (uPA) system, notably PlauR (uPAR; peaking at 2 hrs) and the two major inhibitors of this system, plasminogen activator inhibitor-1 (PAI-1; peaking at 2–4 hrs) and PAI-2 (peaking at 4–6 hrs) were also strongly upregulated by LPA. Through its action on extracellular matrix and cell-surface proteins, the uPA system modulates cell migration and cell-matrix interactions and thereby plays a key role in wound healing, angiogenesis and tumor progression.

Genes encoding additional extracellular mediators and surface-exposed proteins were induced in a second wave of transcription with expression peaking at 4–6 hr after LPA stimulation (Figure 2; see additional files 3 and 4/Table 1). These included the chemokines CCL-2, CCL-7 and Cx3Cl1, the matrix metalloproteinases Adam19 and MMP3, and the transmembrane glycoprotein CD44, which plays a key role in cell-cell interactions. A very strongly upregulated gene was *ILl1rl1*, which encodes an IL-1 receptor family member (murine ST2; ~40-fold induction at 4 hrs). The secreted form of ST2 inhibits the production of cytokines in inflammatory cells [28]. Thus, ST2 upregulation in LPA-stimulated fibroblasts may serve to temper inflammatory responses. Consistent with our data, ST2 was recently identified as major LPA target gene in osteoblastic cells, where it is thought to play an anti-inflammatory role during bone healing [29]. Strong upregulation was also observed for tissue factor (coagulation factor III, encoded by *F3*), a cell-surface glycoprotein that initiates the clotting cascade and has additional roles in cell migration and angiogenesis [30]. Late upregulation (peak expression at ~6 hrs: see additional file 3) was observed for *Lgals3 *and *Timp1*. *Lgals3 *encodes galactin-3, a mediator of inflammation, while secreted Timp-1 has a role in wound healing and the creation of a prometastatic niche.

In conclusion, LPA-stimulated fibroblasts are transcriptionally committed to produce numerous factors known to act on nearby epithelial cells, leukocytes and endothelial cells. LPA stimulation thus enables fibroblasts to promote tissue remodeling, inflammation, angiogenesis, wound healing and, in a tumor context, cancer progression. A schematic representation of the LPA-induced gene expression program over time is shown in additional file 7.

### LPA dose dependence

**Figure 5:**
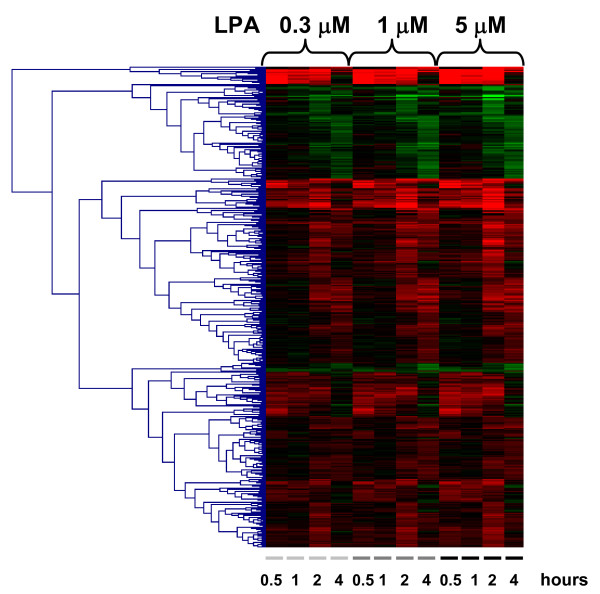
**LPA dose dependence**. The global transcriptional response of quiescent MEFs to different LPA concentrations (0.3, 1.0 and 5.0 μM) at the indicated time points (0.5–4 hrs). For each time point, transcripts with p < 0.01 in 3 out of 4 measurements were selected; the resulting 915 genes were subjected to hierarchical clustering analysis.

**Figure 6:**
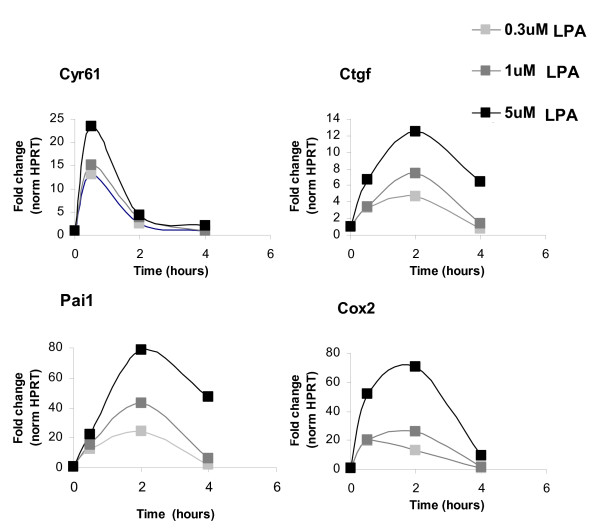
**Temporal expression of selected genes at different LPA concentrations validated by qPCR**. qPCR data were normalized to HPRT mRNA concentration and plotted relative to the level at time zero. Data are presented as means ± SD of duplicate experiments.

**Figure 7:**
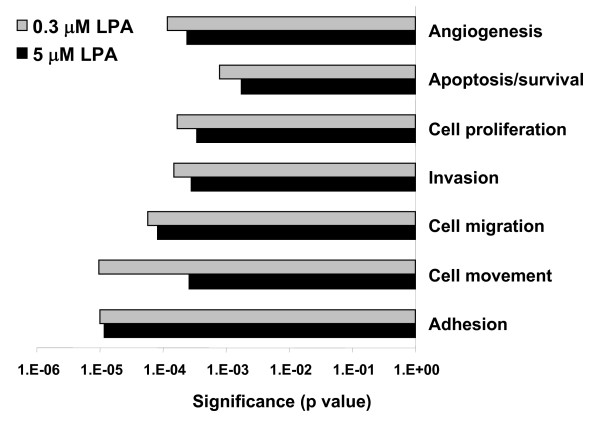
**Ingenuity pathway analysis of gene expression at two different LPA concentrations**. Note relative enrichment of 'cell movement' genes at the lower LPA concentration.

The early cellular responses to LPA, such as cytoskeletal reorganization and migration, usually show their maximal induction in the submicromolar concentration range, while cell cycle progression requires 1–5 μM doses. We determined the dose-efficacy of LPA on gene transcription using three different concentrations (0.3, 1.0 and 5 μM) and analyzed expression profiles over time (0–4 hrs). The regulation of many LPA target genes was preserved at the lowest LPA dose tested (0.3 μM). About 65% of all target genes showed significant regulation by LPA at all three LPA doses (P-values < 0.01; although in many cases the ratios decreased below the threshold of 1.7-fold induction). Increasing the LPA concentration caused increasingly stronger gene expression, often with more prolonged kinetics, as visualized by heat map (Figure 5) and quantitated for selected genes by qPCR (Figure 6). It is of note that many of the genes encoding secreted factors (*Il1rl1, Pai2, Ccl2, Ccl7, Cx3Cl1*, *Hbegf*, *Vegf*) reached their maximal expression already at 0.3 μM LPA. "Ingenuity" pathway analysis indicated that the functional categories modulated by LPA were preserved at all three concentrations, with the notable exception that lowering the LPA dose to 0.3 μM led to a relative enrichment of genes associated with "cell movement" (Figure 7). This result is consistent with LPA's propensity to act as a motility factor and chemo-attractant rather than a growth factor in the lower concentration range.

### Expression profiles of LPA and EGF show broad overlap, but at least 100 genes are differentially regulated

**Figure 8:**
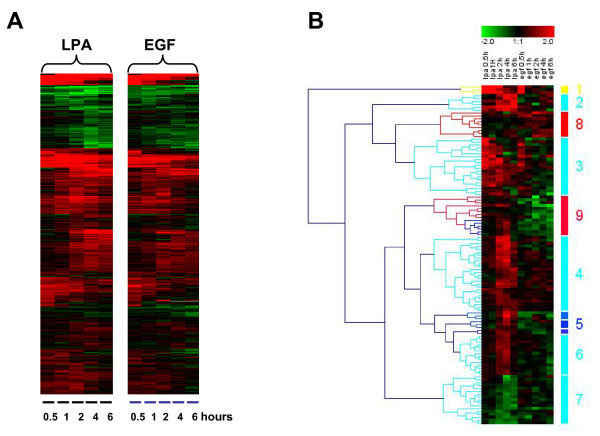
**Differential expression patterns of LPA and EGF**. **(A) **Global transcriptional responses to LPA (5 μM) versus EGF (50 ng/ml) over time. Hierarchical clustering analysis was done on the 915-gene data set of Figure 5. **(B) **Heat map of 111 reporters (encoding 105 genes) that are differentially regulated by LPA (cyan) and EGF (red), or inversely regulated by either agonist (blue). See also additional file 8: heat map of selected genes. Numbers (1–9) refer to additional file 9, which shows a list of all 111 reporters. Genes were identified by Anova analysis (two groups, p < 0.01).

**Figure 9:**
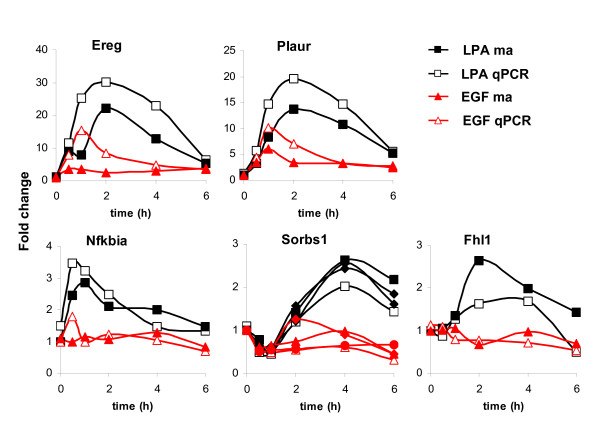
**Differential regulation of selected genes by LPA (black) and EGF (red) validated by qPCR**. qPCR data were normalized to HPRT mRNA concentration and plotted relative to the level at time zero. Data are presented as means ± SD of duplicate experiments.

Fibroblasts have long been used as a model to study peptide growth factor signaling. When stimulated by distinct peptide growth factors (EGF, FGF, PDGF), fibroblasts show a strongly conserved gene-expression signature [31]. This is not too surprising since the cognate receptor tyrosine kinases (RTKs) all use the same signaling principle. To our knowledge, however, it is unknown to what extent the transcriptional response to GPCR stimulation bears comparison with that to RTK stimulation in the same cell type. We therefore compared the temporal gene expression programs of LPA and EGF in MEFs at five different time points (0.5–6 hrs). We found that EGF (20 ng/ml) induced many of the same genes as LPA (5 μM), although LPA stimulation often led to a higher level of induction and/or more prolonged kinetics (Figure 8A,B). For example, LPA caused a much more prolonged upregulation of the immediate early genes *Fos*, *Dusp1 *and *Cxcl1 *than did EGF (Figure 8B; see additional file 8: cluster 1). LPA was also more efficacious in inducing genes that encode paracrine factors (*Ccl2, Ereg, Il1RL1, Ctgf, Vegfa*) and components of the plasminogen activator system (*Plaur, Pai-1*) (Figure 8B). Quantitative PCR analysis confirmed the differential regulation of selected genes by LPA and EGF (Figure 9). A complete list of the differentially regulated genes is shown in additional file 9. To what extent these quantitative differences reflect different expression levels of the respective receptors is currently unknown.

Despite this large overlap of the LPA and EGF expression profiles, approx. 7% of the genes (105 out of 1508 transcripts) was differentially regulated by LPA (Figure 8; see additional files 8 and 9). Immediate-early genes that were upregulated by LPA, but not EGF, include *Edn1 *(endothelin-1), *Fgf16*, *Nfkbia *(NF-kappaB inhibitor alpha) and several protein kinase genes (*Bmp2k*, *Plk2, Tesk2*, *Pim1*), as shown in additional file 8 (cluster 3). Interestingly, LPA also induced the expression of a newly identified GPCR for LPA, termed P2Y5 (encoded by *P2ry5 *[32]), which adds an element of feedback to the fibroblast response to LPA (see additional file 8: cluster 3). At 2–4 hrs, more LPA-specific transcripts could be identified (Figure 8B; see additional files 8 and 9). Gene ontology analysis revealed that the LPA-specific gene set was enriched for genes associated with cytoskeletal organization and integrin signaling, notably those encoding various actin isoforms (*Actb, Acta1, Actg2*), palladin (*Palld*), vinculin (*Vcl*), an Arp2/3 subunit (*Arpc5*), calponins (*Cnn1, Cnn3*), a Rho GTPase (*Rhoj*), Rho-kinase (*Rock2*), myosin X (*Myo10*) and an integrin subunit (*Itga5*) (see additional file 9). Specific upregulation of cytoskeleton-associated genes is in line with LPA's role as an efficacious regulator of cell shape and motility.

**Figure 10:**
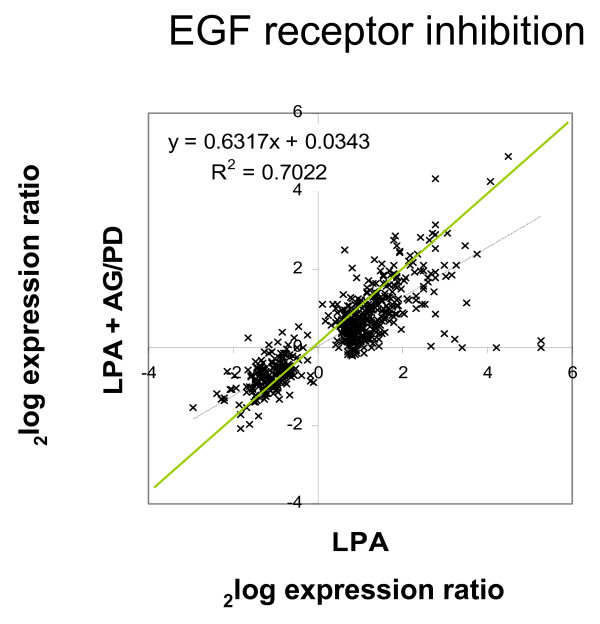
**Correlation plot of the effect of EGF receptor inhibition**. MEFs were treated with a mixture of two EGF receptor inhibitors (AG1478 and PD168393, 250 nM each) or DMSO (control) prior to stimulation with LPA (5 μM) for 4 hrs. Microarray hybridization was performed using the corresponding time-zero control with the same pretreatment. The ^2^log expression level of 528 LPA-regulated genes after 4 hrs is shown as a dot plot to correlate the effect of drug treatment on the expression level of individual genes. The expression level of 528 LPA-regulated genes in control cells was set at 100%. The overall reduction of LPA-induced expression by the inhibitors was approx. 35%, as inferred from the correlation coefficient.

It has long been proposed that GPCR ligands such as LPA signal through 'transactivation' of the EGF receptor [33,34]. According to this model, GPCR agonists rapidly activate the EGF receptor to exploit the tyrosine-posphorylated receptor as a signaling intermediate. However, blocking EGF receptor activity by the selective EGF receptor kinase inhibitor AG1478 (250 nM) had no effect on LPA-induced MAP kinase activation, *Ccl2 *expression and DNA synthesis, while the responses to EGF were fully inhibited (additional file 10 and results not shown). This is in agreement with a previous study showing that LPA mitogenic signaling in MEFs does not require EGF receptor tyrosine phosphorylation [35]. Figure 10 illustrates that the transcriptional response to LPA was only little affected by EGF receptor inhibitor treatment (expression of 528 genes, reproducibly regulated by LPA at three different concentrations at T = 4 hr). About 15% of the LPA-induced genes (81 out of 528 transcripts) was >70% inhibited after drug treatment. Otherwise, EGF receptor inhibition did not affect the induction of key immediate-early and early genes by LPA, such as transcription factors and paracrine mediators. While it remains formally possible that basal EGF receptor activity has a permissive effect on some LPA-induced signaling events, we conclude that LPA and EGF signal independently to regulate broadly overlapping sets of genes in MEFs. It thus appears that the transcriptional program induced by either LPA-GPCR or EGF-RTK stimulation in fibroblasts is more strongly conserved than previously appreciated.

## Conclusion

Dissecting the transcriptional response to growth factors in selected cell systems may help to better understand various aspects of embryonic development, adult tissue homeostasis and cancer. The present study characterizes the global transcriptional program of MEFs to LPA and thereby provides new insights into the normal physiological response of quiescent fibroblasts to this multifunctional lipid mediator. In addition to genes associated with cell proliferation, adhesion and migration, LPA induces a host of genes that encode secreted factors known to promote tissue remodeling, wound healing, inflammation, angiogenesis and tumor progression, depending on cellular context. This highlights the importance of LPA signaling in profoundly modifying the fibroblast microenvironment.

Previous transcriptional profiling of serum-stimulated human skin fibroblasts has identified a 'core serum response' (CSR) that is characterized by cell-cycle-independent genes and reflects various aspects of wound healing, notably the induction of genes involved in matrix remodeling and re-epithelialization [36]. This fibroblast CSR or "wound-response signature" is recapitulated in human carcinomas and may help predict tumor progression [37]. Comparative analysis revealed, however, that the expression profile of LPA-stimulated MEFs shows only limited overlap with the canonical CSR of serum-stimulated human fibroblasts. At first sight, this result is somewhat unexpected since LPA is a major serum constituent [1,13]. On the other hand, serum is an ill-defined mixture of numerous bioactive factors and it is likely that the combined action of many different factors obscures the comparison between serum and LPA. Moreover, MEFs differ from human skin fibroblasts not only in their biological and anatomic origin, but also in their LPA receptor expression pattern (data not shown). Yet, it should be emphasized that LPA-stimulated MEFs and serum-stimulated human skin fibroblasts both show a gene expression profile that is strongly associated with tissue remodeling as well as tumor progression.

Of final note is our finding that the transcriptional response of MEFs to LPA versus EGF shows an overlap of >90%, at least qualitatively, despite the fact that LPA and EGF signal via completely different mechanisms. LPA-induced gene expression was largely independent of EGF receptor activity, which argues against the notion that LPA exploits the EGF receptor as a signaling intermediate. The broad overlap between LPA- and EGF-induced gene expression shows that GPCRs and RTKs have more in common than previously appreciated. One should not conclude, however, that the transcriptional response to receptor stimulation is less dependent on the nature of the receptor than on the cell type. For example, the common expression pattern of LPA and EGF shows hardly any overlap with that induced by Wnt signaling in fibroblasts [38]. The great diversity of fibroblast responses to LPA as reported here is an important area for further study.

## Methods

### Cell culture

Mouse embryonic fibroblasts (MEFs) were immortalized at passage 2 by retroviral introduction of the T-box member Tbx2 (LZRS-TBX2-ires-EGFP) to represses p53 function [39]. MEFs were cultured in Dulbecco's Modified Eagle's Medium (DMEM) supplemented with 8% fetal calf serum (FCS), penicillin and streptavidin, and seeded every three days at a density of 1.6 × 10^4^/cm^2 ^according to the 3T3 protocol.

### RNA isolation and amplification

Detailed protocols for RNA isolation, amplification, labeling and microarray hybridization can be found at . MEFs were seeded at density of 2.5 × 10^4^/cm^2^, and 24 h later were starved for 40 h in serum-free medium supplemented with 0.2% bovine serum albumin (BSA; Sigma). Cells were treated with oleoyl LPA (0.3–5 uM) coupled to fatty acid free BSA (Sigma) at a 3:1 molar ratio, or EGF (20 ng/ml) for the indicated time period. Before harvesting, cells were washed twice with ice-cold phosphate buffered saline. Isolation of total RNA was performed with RNAzol Bee (Campro Scientific, Amersfoort, the Netherlands). Isolated total RNA was subsequently Dnase1-treated by using the Qiagen RNase-free DNase kit (Cat. no. 74106) and RNeasy spin columns (Qiagen, West Sussex, UK, Cat. no. 79254) and dissolved in RNase-free H_2_O. RNA concentration and purity was measured on a NanoDrop ND-1000 spectrophotometer (Isogen Life-Science), while RNA integrity was determined by agarose gel electrophoresis. Four micrograms of total RNA was used to synthesise cDNA with a T7-(dT)24 primer and RT superscript III (Invitrogen Life Technologies; Cat. no. L1016-01). After second-strand synthesis and removal of contaminating RNA, cRNA was generated by *in vitro *transcription using T7 RNA polymerase. Amplification yields were 1,000- to 2,000-fold.

### Microarray processing and data analysis

The cRNA of each sample was labeled with Cy5 or Cy3 (Universal Linkage System, Kreatech Biotechnology, Cat. no. EA-006) according the manufacturer's protocol, with minor adjustments. For each labeling, 0.3 μl of the Cy5-ULS or 1 μl Cy3-ULS was mixed with 1 μg of cRNA and 2 μl of labeling solution. The volume of this mix was adjusted to 20 μl and incubated for 30 minutes at 85°C, followed by purification with a KREA *pure *column. Dye incorporation was determined after measuring the labeled cRNA on a NanoDrop ND-1000 spectrophotometer and mixed with the same amount of reverse color Cy-labeled cRNA from the untreated control. Labeled cRNAs were fragmented to pieces of 60–200 nt (Ambion, Cat. no. AM8740). Before hybridization, 20 μg of COT-1 DNA (Invitrogen-Life Technologies, Cat. no. 15279-011), 8 μg of yeast tRNA (Roche Diagnostics B.V., Cat. no. 10109495001) and 20 μg of poly-d(A) (GE Healthcare Biosciences Europe GmbH, Cat. no. 27-7836-03) was added and the volume was adjusted to 60 μl. To this volume 60 μl of a formamide buffer containing 50% Formamide, 50% 20 × SSC (NaCl/Na-citrate) and 0.1% SDS was added. All hybridizations were performed in a hybridization station (Tecan, Cat. no. Hs4800). Before hybridization with the labeled RNA samples, the microarray was pre-hybridized with a bovine serum albumin solution (1% BSA, 5 × SSC and 0.1% SDS) for one hour at 42°C and washed with water and a 5 × SSC, 0,1% SDS solution. The labeled material was denatured at 95°C for 3 minutes and cooled to 42°C before injection in the hybridization chamber. After hybridization for 16 hrs, slides were washed in the hybridization station with a 5 × SSC, 0,1% SDS solution, a 2 × SSC, 0,1% SDS solution and a 1 × SSC solution at 42°C and a 0,2 × SSC solution at 23°C. Finally, the slides were dried with medical grade nitrogen for 3 min. at 30°C. A DNA Microarray scanner (Agilent Technologies, Cat. no. G2505B) was used to scan the slides. To monitor the consistency of the array experiments, "self-self" experiments were performed using the same sample as reference. Fluorescent intensities of the images were quantified by using ImaGene v6.0 software (Biodiscovery Inc.). This software has an output of two text files which were uploaded to the CMF database (CMFdb, ) for further analysis. The background-corrected intensities from the Cy5 and Cy3 channel were used to calculate log_2_transformed ratios. These ratios were normalized using a lowest fit per subarray [40]. Experiments done in dye-swap fashion were combined to create one dataset on which an outlier analysis was performed. A weighted average ratio and confidence level (P-value) was calculated per gene by a NKI platform adjusted error model [41], which was fine-tuned by self-self hybridizations. Differentially expressed genes between sample and reference were selected based on their P-value (a gene with a P-value < 0.01 is considered an outlier). Genes were selected for further analysis if they had p-values < 0.01 and 2log ratios greater than 0.67 or smaller than -0.67 corresponding to a 1.7-fold change, unless indicated otherwise. To identify structural patterns of gene expression, selected outliers were used for hierarchical clustering using the complete linkage algorithm and for K-means clustering analysis (Euclidian distance) using the GENESIS program [42]. To identify genes that differ between LPA- and EGF induced treatment Anova analysis was applied (two groups) in which the expression values of two consecutive time points were used. "Ingenuity" pathway analysis was used for functional analysis on the set of significantly modulated genes to identify affected biological pathways and functional processes. In addition, the web-based platform Gene Ontology Tree Machine (GOTM) was used to identify GO terms with relatively enriched gene numbers [43].

### Microarray slides

Mouse 32 k Operon v3.0 oligo arrays from the Central Microarray Facility (CMF) at the Netherlands Cancer Institute were used for hybridization. A complete list of genes and controls present on the slides is available on the CMF web site . The 70-mer oligo's (Operon, AROS v3.0) were printed on UltraGaps slides (Corning) with a BioRobotics MicroGrid II (Genomic Solutions) print robot. The description of our microarray study follows the MIAME guidelines and the entire microarray data set has been deposited in the EBI/ArrayExpress database and is accessible through accession numbers E-NCMF-16, E-NCMF-17, E-NCMF-18 and E-NCMF-19).

### Quantitative real-time RT-PCR

Total RNA was extracted using the RNeasy minikit combined with on-column Dnase-I treatment (Qiagen, West Sussex, UK) and resolved in diethyl pyrocarbonate-treated H_2_O (DEPC; Sigma). First-strand cDNA synthesis was done using 2 ug of total RNA in presence of 0.5 ug oligo-dT primers (Invitrogen), 40 units RNase inhibitor Rnasin (Promega), 500 nM deoxynucelotide triphosphates (Roche), 10 uM DTT and 10 units Superscript II RT in 1× reverse transcriptase buffer (Invitrogen). Sequences of real-time quantitative PCR primers were designed using Primer express software (PE Biosystems, Foster City, CA). Primer sequences are available upon request. Detection and quantification of each gene was accomplished by SYBRgreen incorporation using the ABI PRISM 7700 sequence detection system (Applied Biosystems). Quantitative RT-PCR was carried out using 40 ng cDNA, 300 nM of each oligo in presence of 1× SYBRgreen mix in 20 ul reactions (Applied Biosystems). Cycling parameters were: 2 min incubation at 50°C, 10 min. incubation at 95°C, followed by 50 PCR cycles consisting of 15 sec at 95°C and 1 min. at 60°C. Product sizes were routinely verified by collecting a melting curve from 55°C to 95°C after final amplification. The relative product levels were quantified using the 2^-ΔΔCT ^method. Data are presented as relative induction of each target gene, normalized to the expression of HPRT, and are representative of two independent experiments.

## Authors' contributions

CS performed the experiments, did the data and bioinformatics analysis and drafted parts of the manuscript. RK participated in the microarray experiments and helped with data analysis. WHM conceived and coordinated the study and wrote the manuscript. All authors read and approved the final manuscript.

## Supplementary Material

Additional file 1 **Complete data set of LPA-regulated target genes in MEFs**. 1508 genes were selected based on the criteria p < 0.01 in at least two samples (i.e. two different time points or the same time point in independent dose-response experiments; Figure 5), with a minimal fold-change of >1.5. Indicated are the ^2^log ratios. Click here for file

Additional file 2 **Gene expression profiles clustered into different classes: immediate-early and early genes**. See Figure 2 for details. Click here for file

Additional file 3 **Gene expression profiles clustered into different classes: delayed and down-regulated genes**. See Figure 2 for details. Click here for file

Additional file 4 **List of selected genes that are most strongly regulated by LPA (5 μM)**. Excel files showing 141 upregulated genes (Table 1) and 38 downregulated genes (Table 2). Click here for file

Additional file 5 **Correlation plot of qPCR versus MA assays**. Comparison of mRNA levels measured by microarray and qPCR assays. Each data point represents a single gene at a single time point (Y = 0.932x1.24; R2 = 0.8862, R = 0.941). Click here for file

Additional file 6 **Gene ontology analysis of the LPA-induced gene expression program in MEFs**. Functional categories of genes showing peak expression at 2–4 hrs. Click here for file

Additional file 7 **Schematic representation of the LPA-induced expression program over time**. Ingenuity pathway analysis. Red: upregulated genes. Green: downregulated genes. Click here for file

Additional file 8 **Heat map of selected genes that that are differentially regulated by LPA and EGF. **Numbers of the clusters (1–4) refer to those in the heat map of Figure 8B and the list of genes in additional file 9. Click here for file

Additional file 9 **List of genes that are differentially regulated by LPA and EGF over time. **Numbers of the clusters (1–9) refer those in Figure 8B. Genes were identified using Oneway Anova (two groups, p < 0.05, using at least two time points). Click here for file

Additional file 10 **Effect of AG1487 (250 nM) on LPA- and EGF-induced cellular responses. **Upper panel: MAP kinase activation (pERK) as determined by Western blot; tubulin (tub) served as a loading control. Lower panel: Ccl2 mRNA expression after 1 hr of agonist stimulation (qPCR determination). Click here for file
