# Supplementary material for: Multiple actions of lysophosphatidic acid on fibroblasts revealed by transcriptional profiling
Source: BMC Genomics. 2008 Aug 14;9:387. doi: 10.1186/1471-2164-9-387 (PMC2536681; doi:10.1186/1471-2164-9-387)
Supplement: Additional file 2 — Gene expression profiles clustered into different classes: immediate-early and early genes. See Figure 2 for details. [file 1471-2164-9-387-S2.ppt]

## Slide 1
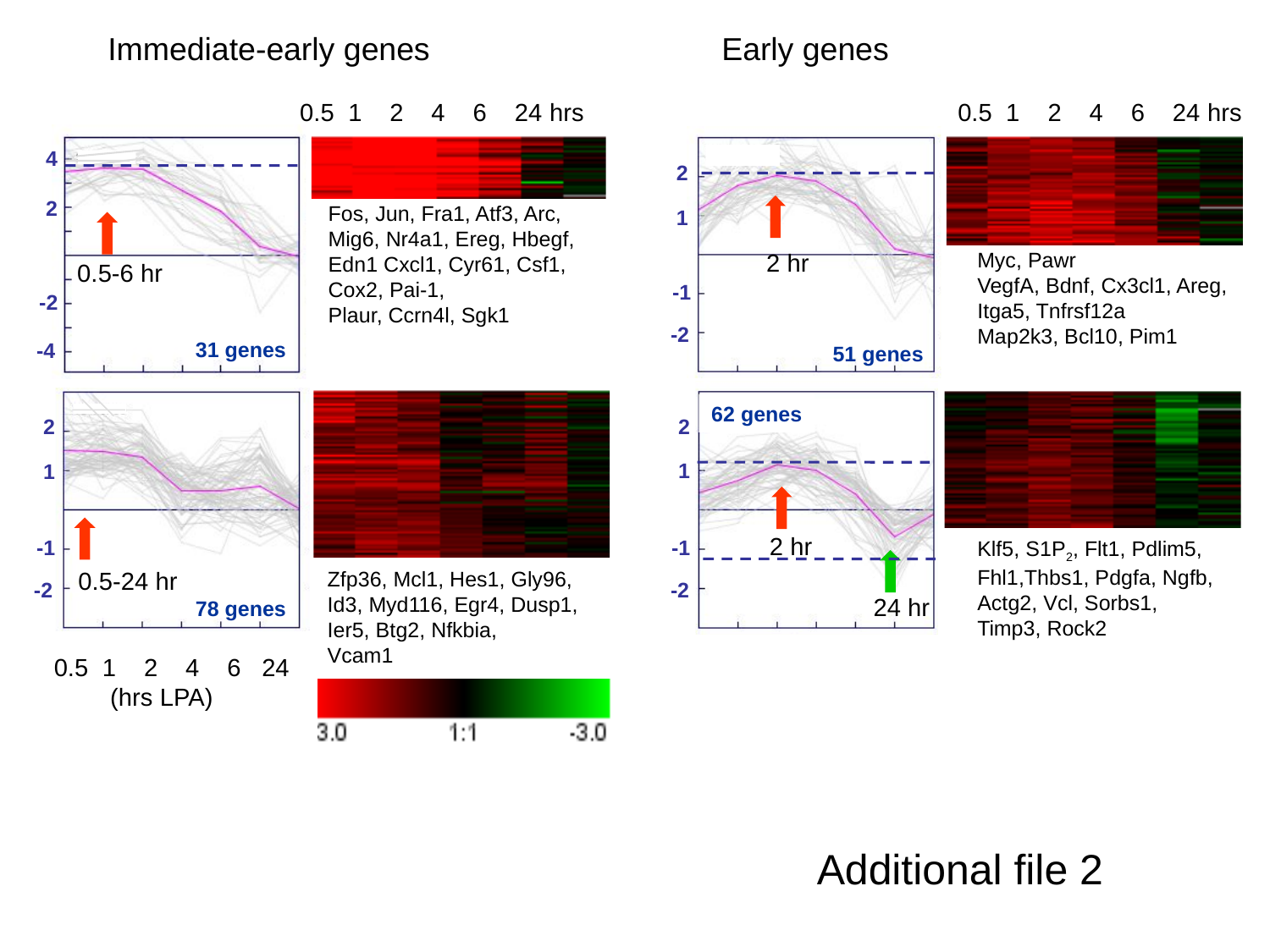

Immediate-early genes
Early genes
0.5 1 2 4 6 24 hrs
0.5 1 2 4 6 24 hrs
4
2
0.5-6 hr
-2
31 genes
-4
2
1
2 hr
-1
-2
51 genes
Fos, Jun, Fra1, Atf3, Arc, Mig6, Nr4a1, Ereg, Hbegf, Edn1 Cxcl1, Cyr61, Csf1, Cox2, Pai-1,
Plaur, Ccrn4l, Sgk1
Myc, Pawr
VegfA, Bdnf, Cx3cl1, Areg, Itga5, Tnfrsf12a
Map2k3, Bcl10, Pim1
2
1
 2 hr
-1
-2
24 hr
62 genes
2
1
-1
-2
0.5-24 hr
78 genes
Klf5, S1P2, Flt1, Pdlim5, Fhl1,Thbs1, Pdgfa, Ngfb, Actg2, Vcl, Sorbs1, Timp3, Rock2
Zfp36, Mcl1, Hes1, Gly96,
Id3, Myd116, Egr4, Dusp1, Ier5, Btg2, Nfkbia,
Vcam1
 0.5 1 2 4 6 24
 (hrs LPA)
Additional file 2
