# Supplementary material for: Multiple actions of lysophosphatidic acid on fibroblasts revealed by transcriptional profiling
Source: BMC Genomics. 2008 Aug 14;9:387. doi: 10.1186/1471-2164-9-387 (PMC2536681; doi:10.1186/1471-2164-9-387)
Supplement: Additional file 3 — Gene expression profiles clustered into different classes: delayed and down-regulated genes. See Figure 2 for details. [file 1471-2164-9-387-S3.ppt]

## Slide 1
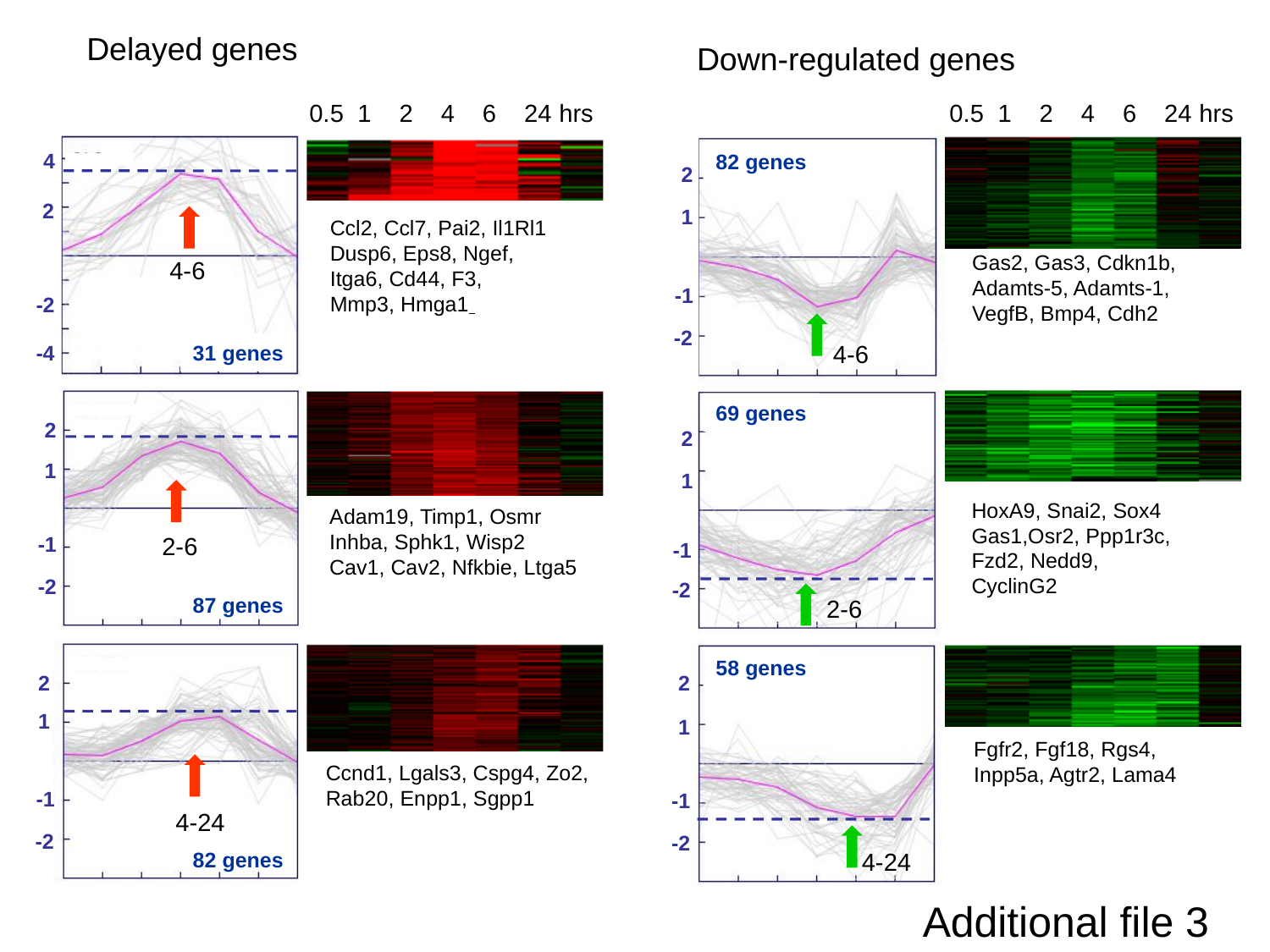

Delayed genes
Down-regulated genes
0.5 1 2 4 6 24 hrs
0.5 1 2 4 6 24 hrs
82 genes
2
1
-1
-2
 4-6
4
2
4-6
-2
-4
31 genes
Ccl2, Ccl7, Pai2, Il1Rl1
Dusp6, Eps8, Ngef,
Itga6, Cd44, F3,
Mmp3, Hmga1
Gas2, Gas3, Cdkn1b,
Adamts-5, Adamts-1,
VegfB, Bmp4, Cdh2
2
1
-1
-2
2-6
69 genes
2
1
-1
2-6
-2
87 genes
HoxA9, Snai2, Sox4
Gas1,Osr2, Ppp1r3c,
Fzd2, Nedd9,
CyclinG2
Adam19, Timp1, Osmr
Inhba, Sphk1, Wisp2
Cav1, Cav2, Nfkbie, Ltga5
2
1
-1
4-24
-2
82 genes
2
1
-1
-2
4-24
58 genes
Fgfr2, Fgf18, Rgs4, Inpp5a, Agtr2, Lama4
Ccnd1, Lgals3, Cspg4, Zo2,
Rab20, Enpp1, Sgpp1
Additional file 3
