# Supplementary figures and images for: Multiple actions of lysophosphatidic acid on fibroblasts revealed by transcriptional profiling
Source: BMC Genomics. 2008 Aug 14;9:387. doi: 10.1186/1471-2164-9-387 (PMC2536681; doi:10.1186/1471-2164-9-387)

## Slide 1
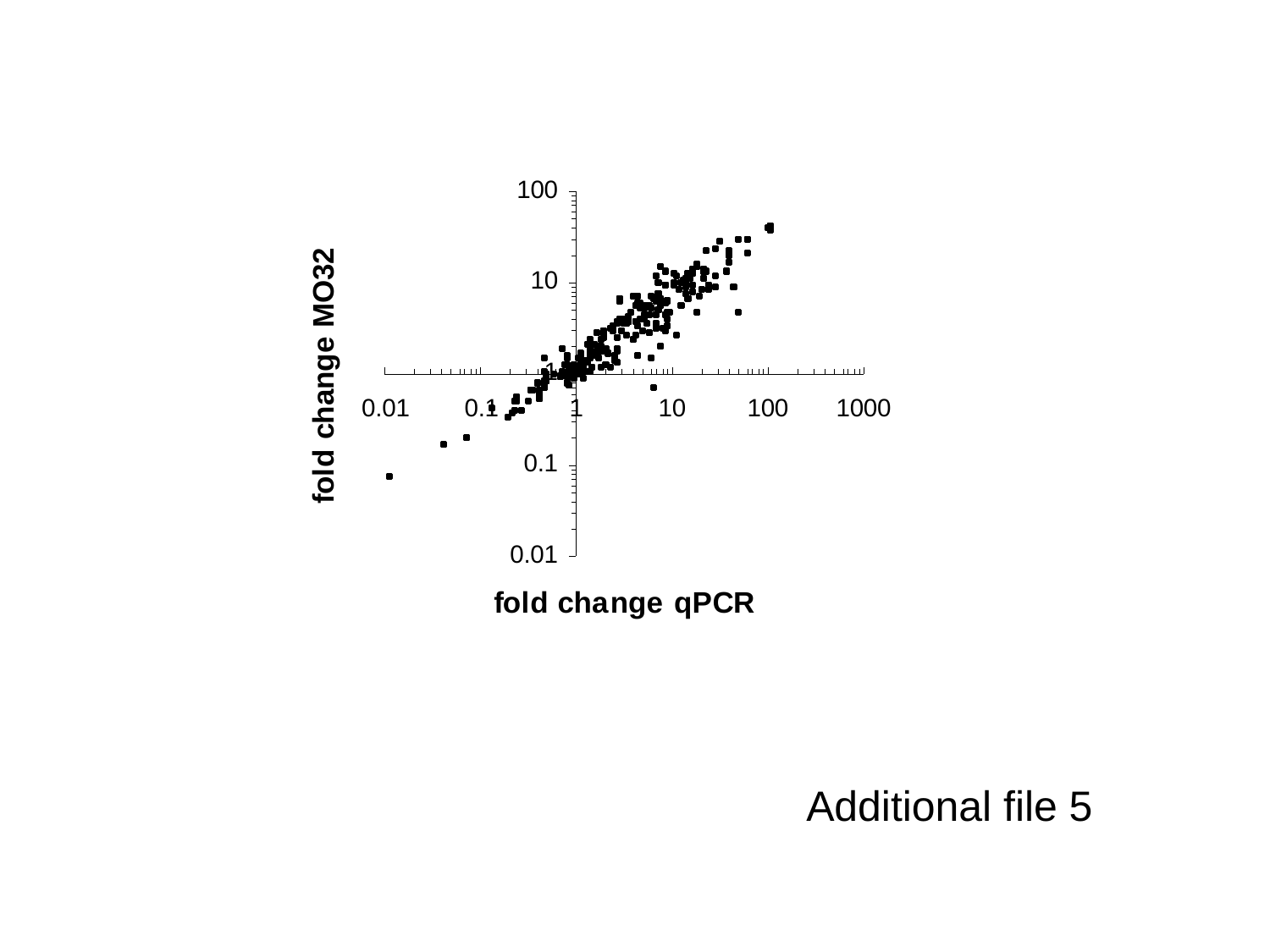

Additional file 5

Supplement: Additional file 5 — Correlation plot of qPCR versus MA assays. Comparison of mRNA levels measured by microarray and qPCR assays. Each data point represents a single gene at a single time point (Y = 0.932x1.24; R2 = 0.8862, R = 0.941). [file 1471-2164-9-387-S5.ppt]

## Slide 1
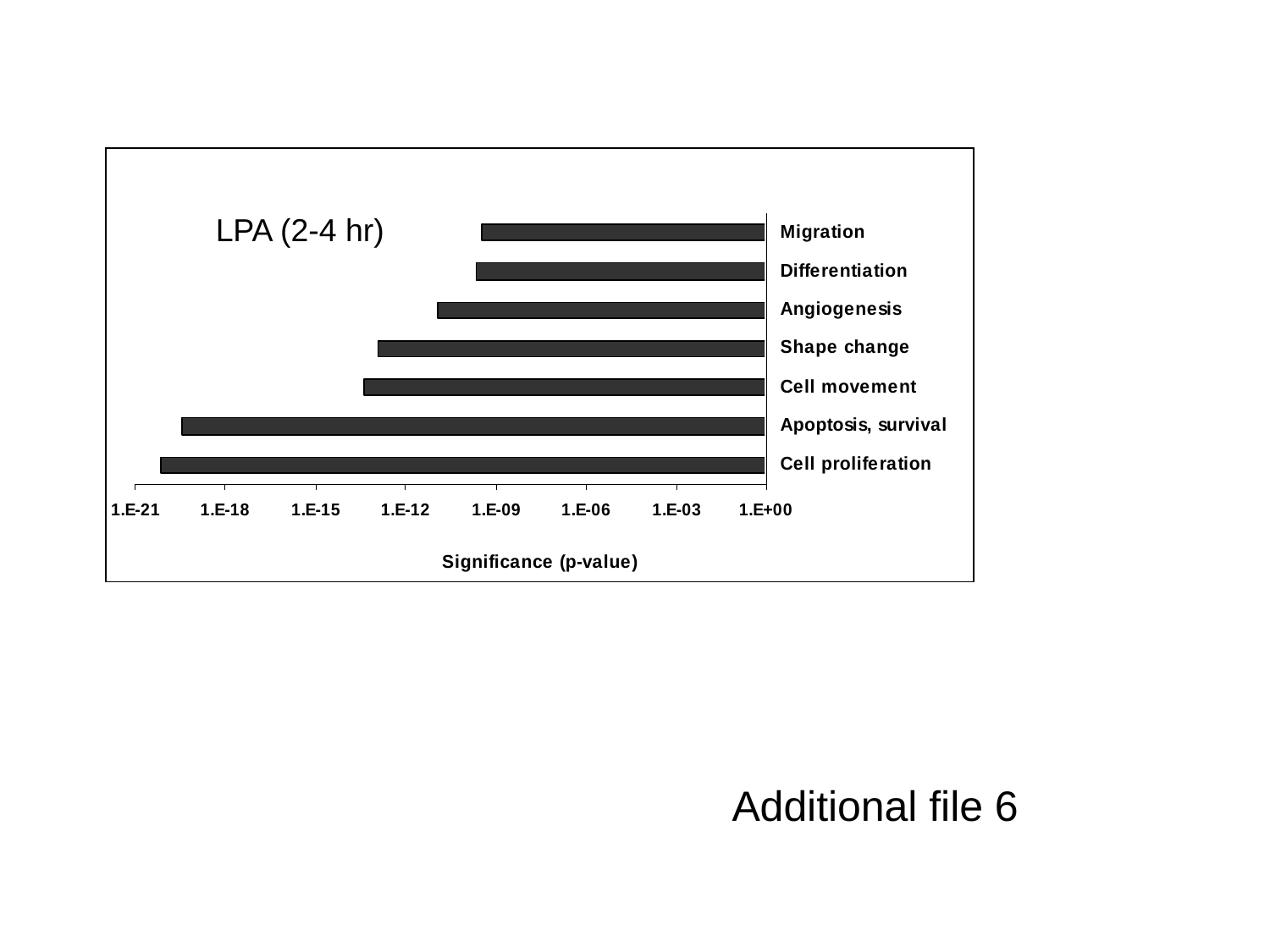

LPA (2-4 hr)
Additional file 6

Supplement: Additional file 6 — Gene ontology analysis of the LPA-induced gene expression program in MEFs. Functional categories of genes showing peak expression at 2–4 hrs. [file 1471-2164-9-387-S6.ppt]

## Slide 1
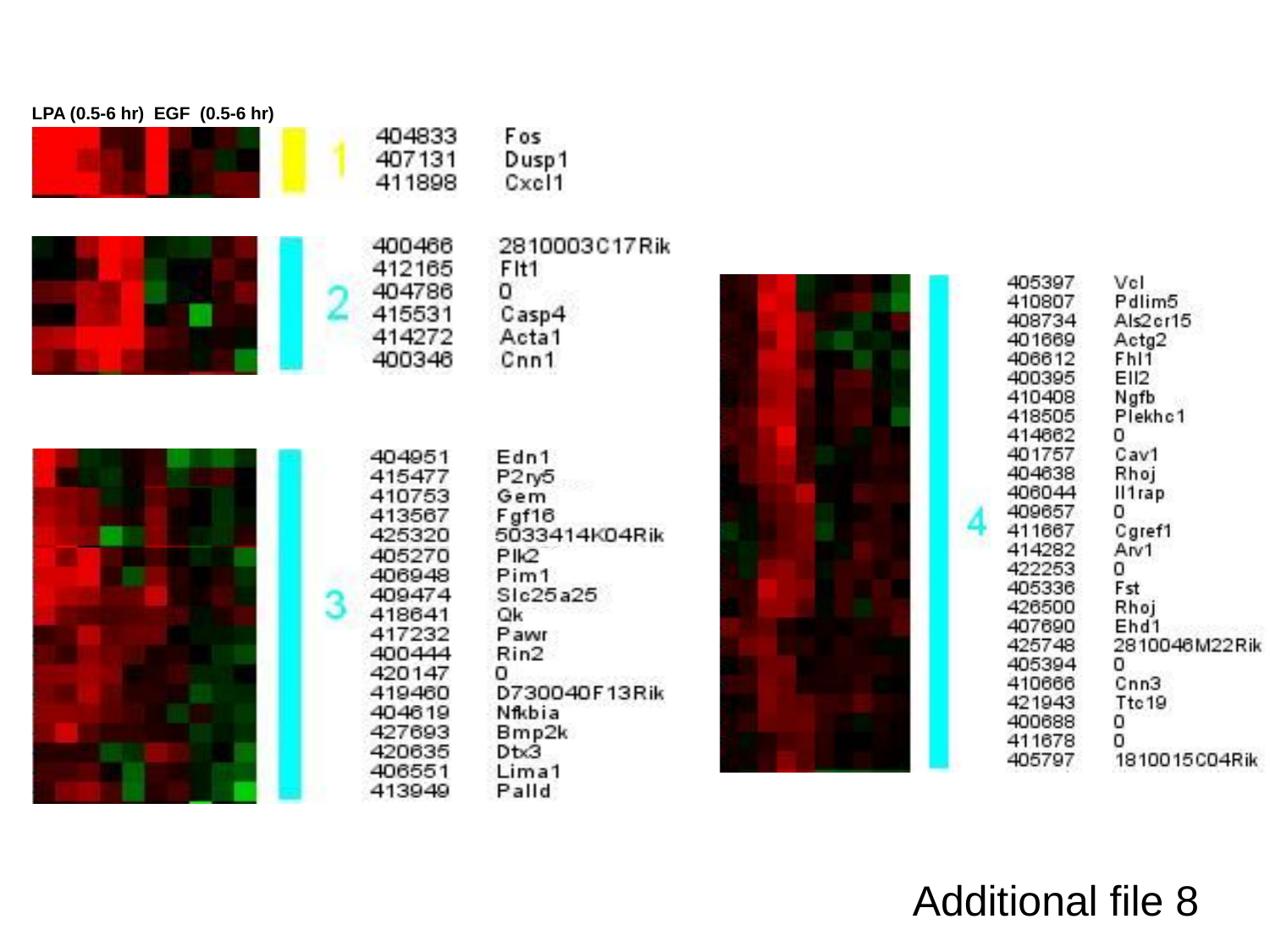

LPA (0.5-6 hr) EGF (0.5-6 hr)
Additional file 8

Supplement: Additional file 8 — Heat map of selected genes that that are differentially regulated by LPA and EGF. Numbers of the clusters (1–4) refer to those in the heat map of Figure 8B and the list of genes in additional file 9. [file 1471-2164-9-387-S8.ppt]

## Slide 1
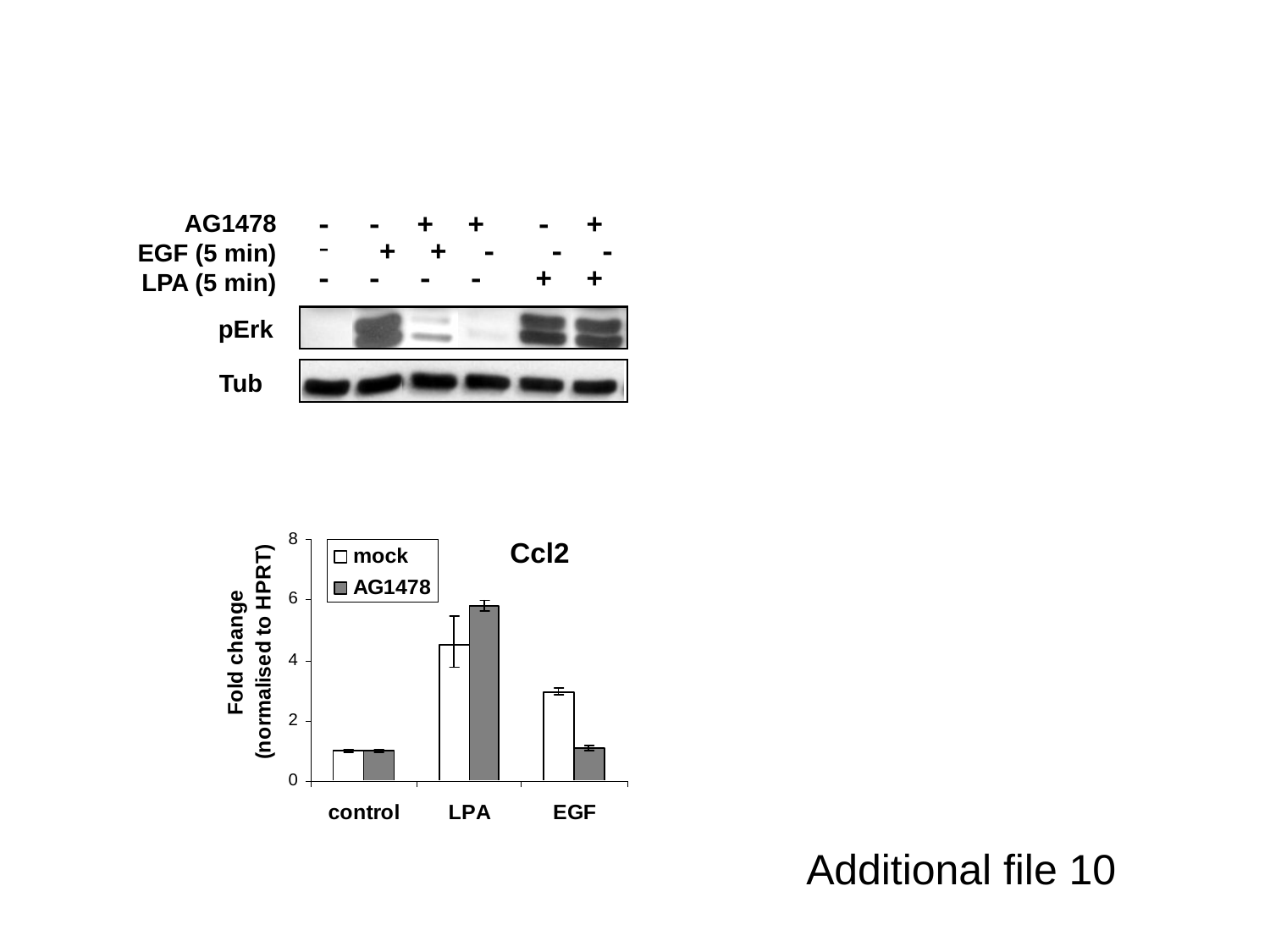

AG1478
EGF (5 min)
LPA (5 min)
- - + + - +
 + + - - -
- - - - + +
pErk
Tub
Ccl2
Additional file 10

Supplement: Additional file 10 — Effect of AG1487 (250 nM) on LPA- and EGF-induced cellular responses. Upper panel: MAP kinase activation (pERK) as determined by Western blot; tubulin (tub) served as a loading control. Lower panel: Ccl2 mRNA expression after 1 hr of agonist stimulation (qPCR determination). [file 1471-2164-9-387-S10.ppt]
