# Supplementary material for: Multiple actions of lysophosphatidic acid on fibroblasts revealed by transcriptional profiling
Source: BMC Genomics. 2008 Aug 14;9:387. doi: 10.1186/1471-2164-9-387 (PMC2536681; doi:10.1186/1471-2164-9-387)
Supplement: Additional file 7 — Schematic representation of the LPA-induced expression program over time. Ingenuity pathway analysis. Red: upregulated genes. Green: downregulated genes. [file 1471-2164-9-387-S7.ppt]

## Slide 1
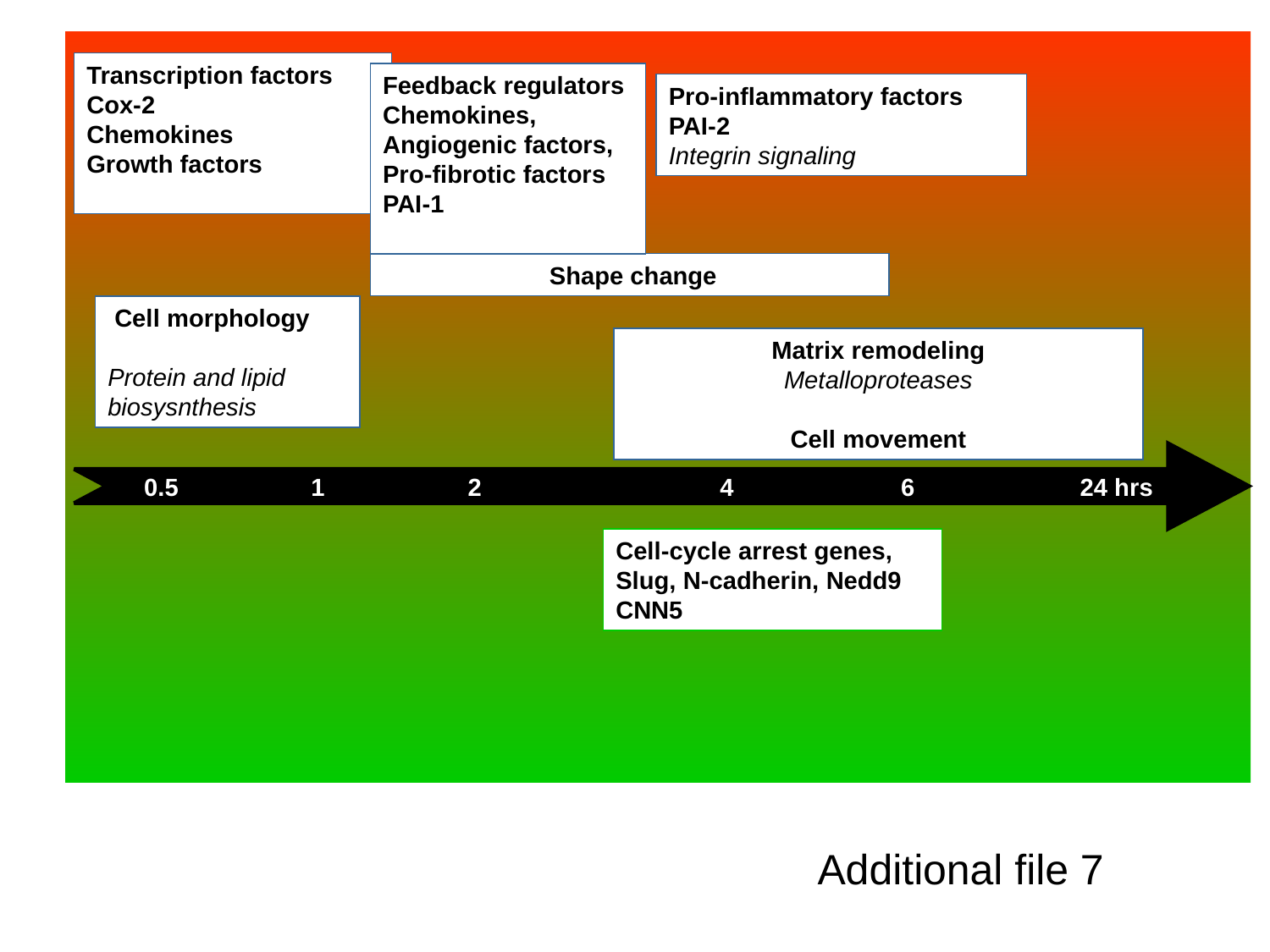

Transcription factors
Cox-2
Chemokines
Growth factors
Feedback regulators
Chemokines,
Angiogenic factors,
Pro-fibrotic factors
PAI-1
Pro-inflammatory factors
PAI-2
Integrin signaling
 Shape change
 Cell morphology
Protein and lipid biosysnthesis
Matrix remodeling
Metalloproteases
Cell movement
 4-24 hrs
 0.5 1
2
 4 6		24 hrs
Cell-cycle arrest genes,
Slug, N-cadherin, Nedd9
CNN5
						Additional file 7
